# Transcription-metabolism association analysis of molecular mechanisms in sweet orange plants in response to boron deficiency

**DOI:** 10.1186/s12864-025-12408-w

**Published:** 2025-12-22

**Authors:** Xiuyao Yang, Ke Wen, Xiujia Yang, Mengjie Zhang, Ling Zhu, Yinqiang Zi, Tuo Yin, Xulin Li, Xiaozhen Liu, Hanyao Zhang

**Affiliations:** 1 Key Laboratory for Forest Resources Conservation and Utilization in the Southwest Mountains of China, Ministry of Education, Southwest Forestry University, Kunming, 650224 China; 2 Key Laboratory of Biodiversity Conservation in Southwest China, National Forest and Grassland Administration, Southwest Forestry University, Kunming, 650224 China

**Keywords:** Sweet orange, *BBX* genes, Boron homeostasis regulation, Transcriptome, Metabolome, Flavonoid biosynthesis

## Abstract

Sweet orange (*Citrus sinensis*) is the most widely grown citrus fruit worldwide, with the highest production and largest cultivated area. Serious problems associated with boron (B) deficiency are emerging in most old production areas, resulting in the lignification of sweet orange flesh and fruit souring. However, the molecular mechanisms through which sweet orange plants react to boron deficiency stress remain unreported in the literature. Through transcriptome analysis and RT-qPCR expression validation, this study revealed that *CsBBX11*, *CsBBX13*, and *CsBBX14* may play important roles in the response of sweet orange plants to boron deficiency stress. Metabolomic analysis revealed that the contents of quercetin 3-glucoside, taxifolin, and isovitexin all exceeded 1 µg/g, with significant differences in the contents of these metabolites observed before and after treatment. Correlation analysis revealed that *CsBBX14* was positively correlated with quercetin 3-glucoside and isovitexin, but negatively correlated with taxifolin. Furthermore, the expression of CsBBX14 was highly significantly correlated with that of 57% of the genes associated with flavonoid biosynthesis and metabolism. Finally, a possible regulatory mechanism for sweet orange resistance to boron deficiencywas proposed in this study. *CsBBX14* is likely to interact with the G-box element located in the promoter region of HY5, thereby initiating the expression of genes responsible for flavonoid synthesis. In conclusion, *CsBBX14* is likely a key gene involved in the synthesis of flavonoids during stress caused by a lack of B. However, its specific functions in flavonoid synthesis need to be further verified. These findings offer a novel reference point for the molecular breeding of sweet oranges aimed at addressing boron deficiency stress.

**Supplementary Information:**

The online version contains supplementary material available at 10.1186/s12864-025-12408-w.

## Introduction

Sweet orange (*Citrus sinensis*) is a fruit tree belonging to the Rutaceae family, specifically within the Citrus genus, and is recognized as the most extensively cultivated and commercially significant fruit species globally. Citrus plants are grown on 143 million acres worldwide, and their production has reached 184 million tons [1]. Fruit is rich in various vitamins, carotenoids, dietary fibers, flavonoids, phenolic compounds, and other bioactive substances [2]. In recent years, the progressive expansion of sweet orange cultivation, coupled with escalating anthropogenic degradation of ecological habitats, has induced a critical deficiency in essential nutrient availability. This nutritional constraint has emerged as a limiting factor for the sustainable intensification of citrus production systems, consequently resulting in quantifiable declines in both fruit yield and qualitative attributes. Boron (B) exists in the soil as boric acid, which tends to leach from the soil during high rainfall because of its high solubility and mobility, which results in soils being highly susceptible to B deficiency problems [3]. B deficiency stunted plant growth by inhibiting root growth, leaf necrosis, and starch accumulation in the body as well as by inhibiting sugar uptake and transport. This results in fleshiness and reduced sweetness and sourness of the fruit, severely affecting fruit quality and yield [4].

B is a crucial micronutrient for plant growth and development, ensuring optimal growth and productivity, which is economically vital. *B-box* (*BBX*) proteins are zinc-finger transcription factors characterized by B-box structural domains. *BBX* genes have one or two conserved B-box structural domains at the N-terminal end, and some BBX proteins also contain CCT structural domains at the C-terminal B-box domains; these domains are associated with interactions between specific proteins, and CCT domains play a regulatory role in gene transcription [5]. Plant B nutrition is closely related to the expression of BBX genes. A study by Li et al.. revealed that under low-boron treatment, six *BnaBBX* genes were significantly upregulated and nine *BnaBBX* genes were upregulated in the roots of rapeseed (*Brassica napus*); in the shoots of rapeseed, three *BnaBBX* genes were significantly downregulated, whereas 3 *BnaBBX* genes were significantly upregulated. These findings indicate that under B deficiency stress, *BBX* genes exhibit differential expression across different plant tissues [6]. *BBX* genes play vital roles in mediating photomorphogenesis and the accumulation of flavonoids. *AtBBX21* and *AtBBX22* in *Arabidopsis thaliana* (*A. thaliana*) interact with and increase the activity of *AtHY5*, promoting the expression of flavonoid biosynthetic genes. It directly activates *AtCH1* transcription to increase anthocyanin accumulation [7]. *MdBBX22* and *MdBBX20* work together with *HY5* to trigger the expression of *MdMYB1*, leading to increased anthocyanin levels in apple plants [8]. The ability of plants to eliminate free radicals is crucial for their resistance systems. Flavonoids directly scavenge excess free radicals, especially ROS, and indirectly scavenge free radicals by enhancing the performance of antioxidant enzymes [9]. Research indicates that naringenin greatly enhances the activities of SOD, CAT, POD, and APX in plants, increasing their capacity to eliminate free radicals [10].

As many species are rapidly sequenced, the importance of transcriptomics and metabolomics in understanding gene functions is increasing [11, 12]. Environmental factors can modulate gene expression by impacting transcription factors, which selectively attach to the promoters of their corresponding target genes, thereby affecting the accumulation of metabolites within the plant. Wen et al. [13] employed a combined transcriptomic and metabolomic analysis to demonstrate that the CCO gene regulates carotenoid content, thereby altering the color of Orah peels. Researchers have employed multiomics analysis to investigate the complex response mechanisms of two alfalfa varieties (ZD and BM) to combined cold and saline_alkali stress, revealing that the *MsMYB12* gene may counteract integrated stress by regulating the biosynthesis pathway of flavonoid compounds [14]. Through comprehensive screening and analysis of rapeseed genes and metabolites under saline_alkali stress via transcriptomics and metabolomics techniques, Ma et al. has elucidated the molecular mechanisms and metabolic pathway alterations underlying the response of rapeseed to saline-alkali stress [15]. These findings may lead to further research on crop breeding and stress tolerance mechanisms.

In this study, the fundamental functions and characteristics of the *BBX* genes were understood through phylogenetic analysis of the sweet orange genome (v3.0), analysis of cis-acting_elements, chromosomal location, and bioinformatics analysis via GO and KEGG. The expression of these genes was determined via transcriptomic analysis combined with flavonoid metabolite analysis to reveal the regulatory mechanism of sweet oranges in response to B deficiency stress and to propose a regulatory model of flavonoid resistance to B deficiency stress in sweet oranges. This study serves as a valuable reference for future research exploring the mechanisms through which boron deficiency influences both the yield and quality of sweet orange fruits.

## Materials and methods

### Plant material treatment

All the samples were obtained from the planting base in Yuxi city, Yunnan Province, China, and the test variety was Bingtang Citrus sinensis. We selected fruits with uniform orange‒yellow skin, removed the pulp, and extracted twenty healthy seeds as materials for subsequent research. The selected seeds were rinsed under running water for one hour and then transferred to modified MS (4.4 mg/L MS + 4.5 mg/L agar + 30 g/L sucrose + 0.8 mg/L IBA) media to be cultured into complete plants. The plant shoots were used to induce callus tissue, and the sweet orange histocultured plants were cultivated through the histoculture system of adventitious shoot induction, rooting induction, and seedling strengthening culture. The seedlings were subsequently transferred to the MS nutrient mixture. The plants were subsequently grown in 0.01 mg/L boric acid medium for boron deficiency treatment, and a boric acid concentration of 0.25 mg/L was used as the control [16]. After 25 days of boron deficiency treatment, significant boron deficiency symptoms were observed in sweet orange plants. Leaves from sweet orange plants were collected, immediately frozen in liquid nitrogen, and then stored at −80 °C for preservation.

### Identification of *BBX* gene family members in sweet orange

Initially, the genomic data associated with sweet orange plants were retrieved from the CPBD database (http://citrus.hzau.edu.cn/). The HMM model of the structural domain (PF00643) was obtained from the Pfam (https://pfam.xfam.org/) database as a query condition [17], and the members of the *BBX* gene family, as identified by HMMER version 3.3.2, were filtered out of the family member sequences with E values < 10^− 5^. Thirty *BBX* gene sequences of *A. thaliana* were subsequently retrieved from the TAIR database (https://www.Arabidopsis.org/). The BLASTP program was used to perform homology comparisons of the whole genome of sweet orange using the *BBX* gene sequence of *A. thaliana* as a reference, after which the gene sequences with an E value < 1e^− 5^ were screened [18]. The screening outcomes were then assessed in conjunction with the results obtained from the HmmSearch program to ascertain the members of the *BBX* gene family. By integrating the findings from both screenings, we eliminated any duplicated or redundant protein sequences, ultimately leading to the identification of the final *BBX* gene family members.

### Phylogenetic analysis

Multiple sequence comparisons of *BBX* genes were performed via the MAFFT program under default parameters [19]. The chromosomal positions of the sweet orange *BBX* genes were established using the Gene Location program in TBtools. Consequently, the naming of these genes was modified to reflect their respective locations on the chromosome. The members of the sweet-orange *BBX* gene family were categorized based on the classification of the *BBX* gene family in *A. thaliana*. To construct the phylogenetic trees, we employed iqtree v1.6.12 [20], which determined the optimal model to be JTT + I + G4, supported by a bootstrap value of 1000.

### Analysis of cis-acting elements in the *BBX* gene family in sweet orange

The 2000 bp CDS located upstream of the gene was extracted from the sweet orange genome GFF file via TBtools. Cis-acting elements were identified via the online program PlantCARE (https://bioinformatics.psb.ugent.be/webtools/plantcare/html/) and subsequently visualized via ChiPlot software [21].

### Chromosomal location, duplicate genes, and covariance analysis

The chromosomal location information for the target genes was obtained from the GFF files and whole-genome sequences of sweet orange via TBtools [22]. The intraspecific covariance of sweet orange *BBX* genes and their duplicate genes was analyzed usingthe MCScanX [23]. Visual analyses were conducted via the Circos program [24]. GFF files and gene sequence files for *Vitis vinifera (V. vinifera)* and *A thaliana* were obtained from the CPBD sweet orange database and the TAIR database, respectively, to establish interspecific covariates.

### Transcriptome analysis and flavonoid content determination

A total of 0.5 g of each sample from the treatment and control groups was sent to Suzhou Panomics Biomedical Technology Co., Ltd., for transcriptome sequencing to obtain the transcriptome data of sweet orange plants under B deficiency stress. The transcriptome sequencing data were processed via fastp to remove the sequences with a splice at the 3′ ends and to remove the reads with an average quality score lower than Q20; the filtered reads were compared to the reference genome via HISAT2 (http://ccb.jhu.edu/software/hisat2/index.shtml). The data quality was checked via FastaQC to ensure that all the transcriptome data had Q values greater than 30, resulting in high-quality data. The read counts were statistically aligned to each gene via HTSeq and normalized to the FPKM values. Differential expression analysis between sample groups was performed via DESeq [25] to obtain the genes differentially expressed between the two biological treatments, from which genes significantly differentially expressed with a fold change ≥ 2 and a P value ≤ 0.05 were screened.

For flavonoid-targeted quantification, for the configuration of standards, the flavonoid standards were accurately weighed, 80% methanol was used to prepare the single standard mother liquor, the appropriate amount of each mother liquor was measured to mix the mother liquor, and 80% methanol was used to dilute, one by one, to a suitable concentration. The stock solution and working solution were stored at −20 °C. For metabolite extraction, an appropriate amount of sample was added to a 2 mL centrifuge tube, 600 µL of methanol was added, the mixture was vortexed for 60 s, and then two steel balls were added. The samples were placed into a tissue grinder and ground at 60 Hz for 1 min. The above procedure was repeated at least twice. The mixture was sonicated for 15 min at room temperature and centrifuged at 12,000 rpm for 5 min at 4 °C. The supernatant was passed through a 0.22 μm filter membrane, the filtrate was added to the assay vial, and the metabolite content was measured on the instrument.

### Quantitative real-time PCR analysis

To further verify the expression of the *CsBBX11*, *CsBBX13*, and *CsBBX14* genes in sweet orange plants under B deficiency stress, at least three replicates were performed for each gene. Total RNA was extracted from the samples via a Genomic RNA Extraction Kit (DP320) and reverse transcribed into cDNA via a FastKing RT Kit (KR116). The reverse transcribed cDNA was mixed with a 10-fold dilution mixture and mixed well for quantitative PCR. The RT-qPCR primers were designed using Primer 6.0, with actin (Gene ID: *Cs_ont_3g025170*) as the internal reference gene (Supplementary Material 1). Standard RT-qPCR was then performed via the SYBR Green RT-qPCR Master Mix Kit, with procedures strictly adhering to the product manual. SPSS v21 was used for data analysis and significant difference analysis [11].

## Results

### Sweet orange *BBX* gene family identification and phylogenetic analysis

**Fig. 1:**
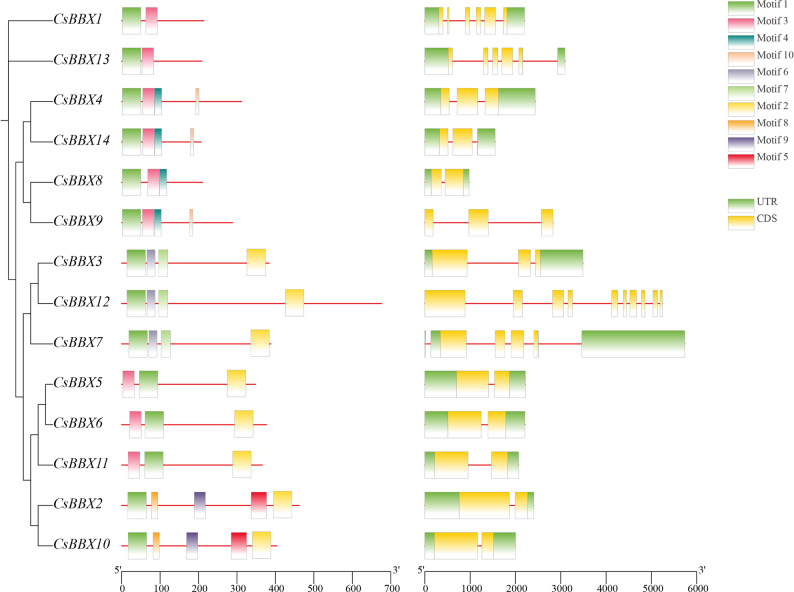
Conserved motif composition and gene structure of sweet orange *BBX* genes

**Fig. 2:**
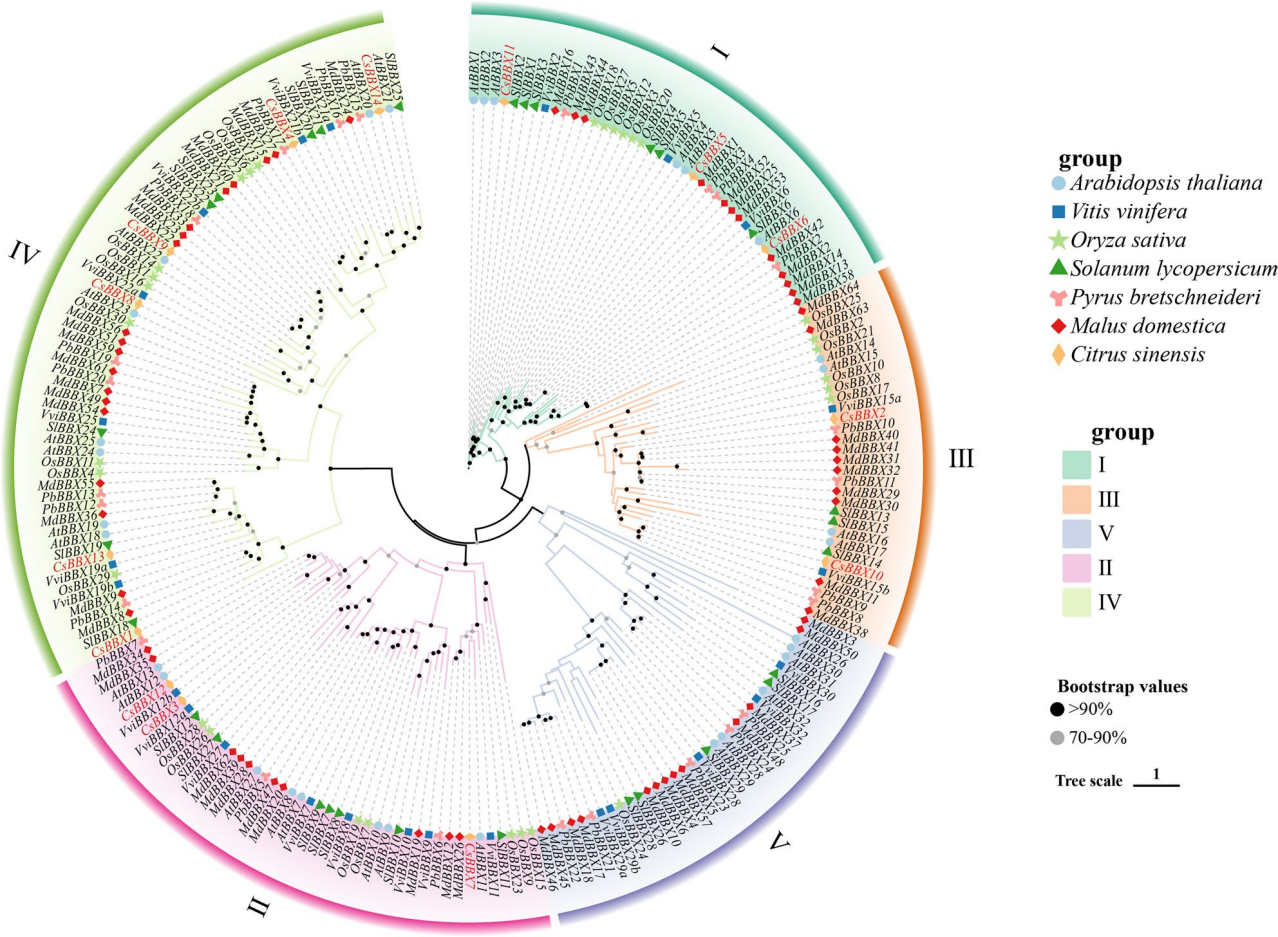
Phylogenetic analysis of *Citrus sinensis*, *Vitis vinifera*, *Arabidopsis thaliana*, *Solanum lycopersicum*, *Malus pumila*, *Pyrus bretschneideri*, and *Oryza sativa BBX* genes. The tree was divided into five branches, I, II, III, IV, and V, marked with different colors. Different species are represented by various symbols

The sequences of 14 members of the sweet orange *BBX* gene family were identified via HMMER and BLAST methodologies and subsequently renamed based on their chromosomal positions (Supplementary Material 2). Based on the conserved structural domains, the *BBX* genes identified in sweet orange were categorized into five distinct groups (types I, II, III, IV, and V) following the classification framework established for the *BBX* gene sequences of *A. thaliana*. The conserved structural domains observed in groups I and II were similar, comprising three distinct elements: B-box1, B-box2, and CCT. In contrast, the members of group III encompassed the B-box1 and CCT domains. Group IV was characterized by the presence of both B-box1 and B-box2, whereas group V was notably defined solely by the existence of a single B-box1 structural domain (Fig. 1). The 14 *BBX* gene sequences of sweet orange and the *BBX* gene sequences of *A. thaliana*, *V. vinifera*, rice, *Solanum lycopersicum*, *Pyrus bretschneideri*, and *Malus pumila* were compared via multiple sequence alignment, the numbers of *BBX* genes in these species were 31, 25, 30, 29, 25, and 64, respectively. A phylogenetic tree was constructed via IQ-TREE v1.6.12 (Fig. 2).The clustering results revealed that subgroup IV contained the greatest number of sweet orange *BBX* genes (6) and that subgroup V had no sweet orange *BBX* genes. The *BBX* genes from the other six species were categorized into five distinct subclades, although the number of genes distributed in each subclade was diverse. These findings indicate that the phylogenies of sweet orange and the other seven species are relatively similar. All 14 *BBX* genes of sweet orange clustered on the same branch as dicotyledons in the phylogenetic tree. Among them, 57% of the genes clustered with *A. thaliana*. These findings indicate that, compared with that of rice (monocotyledonous), the sweet orange phylogeny of these plants is more similar to that of dicotyledonous plants, especially *A. thaliana*.

### Cis-acting element analysis

Cis-acting elements associated with the *CsBBX* genes were identified via PlantCARE to elucidate their functional and regulatory roles. A total of 329 cis-acting elements were identified and classified into four distinct categories (Fig. 4A-B). The plant growth and development response elements included phloem tissue regulatory elements (CAT-boxes), circadian regulatory elements (circadians), endosperm expression regulatory elements (GCN4 motifs), and maize protein metabolism regulatory elements (O2 sites). Among them, the number of CAT-Box (32%) elements related to phloem tissue expression and O2 site (45%) elements involved in maize protein metabolism regulation accounted for a greater proportion of the total number of genes. The plant hormone response elements included the following: ABA response element (ABRE), MeJA response element (CGTCA motif, TGACG motif), partial light response element (P-box), gibberellin-responsive response element (TATC-box), salicylic acid response element (salicylic acid) (TCA-element), auxin-responsive element (TGA-element), and partial light-responsive element (AE-box, ATCT-motif, Box 4). Among them, ABA, MeJA, gibberellin, and salicylic acid are important phytohormones for plants to resist stress, and their related response elements account for 25%, 22%, 2%, and 4%, respectively. The light response elements included the GA motif, GATA motif, I-box, TCCC motif, TCT motif, GT1 motif, Sp1, G-box, and MRE. Abiotic and biotic stress elements include gibberellin (GA)-responsive elements (AREs), anaerobically induced response elements (GC motifs), low-temperature responsive cis-acting elements (LTRs), and drought-induced MYB-acting elements (MBSs) involved in defense and stress responses (TC-rich repeats). The proportions of the abiotic and biotic stress components were 38%, 5%, 12%, 21% and 24%, respectively. The gibberellin (GA) ARE-acting element was the most abundant at 38%. Notably, the number of abiotic and biotic stress elements was six for both *CsBBX11* and *CsBBX13*.

**Fig. 3:**
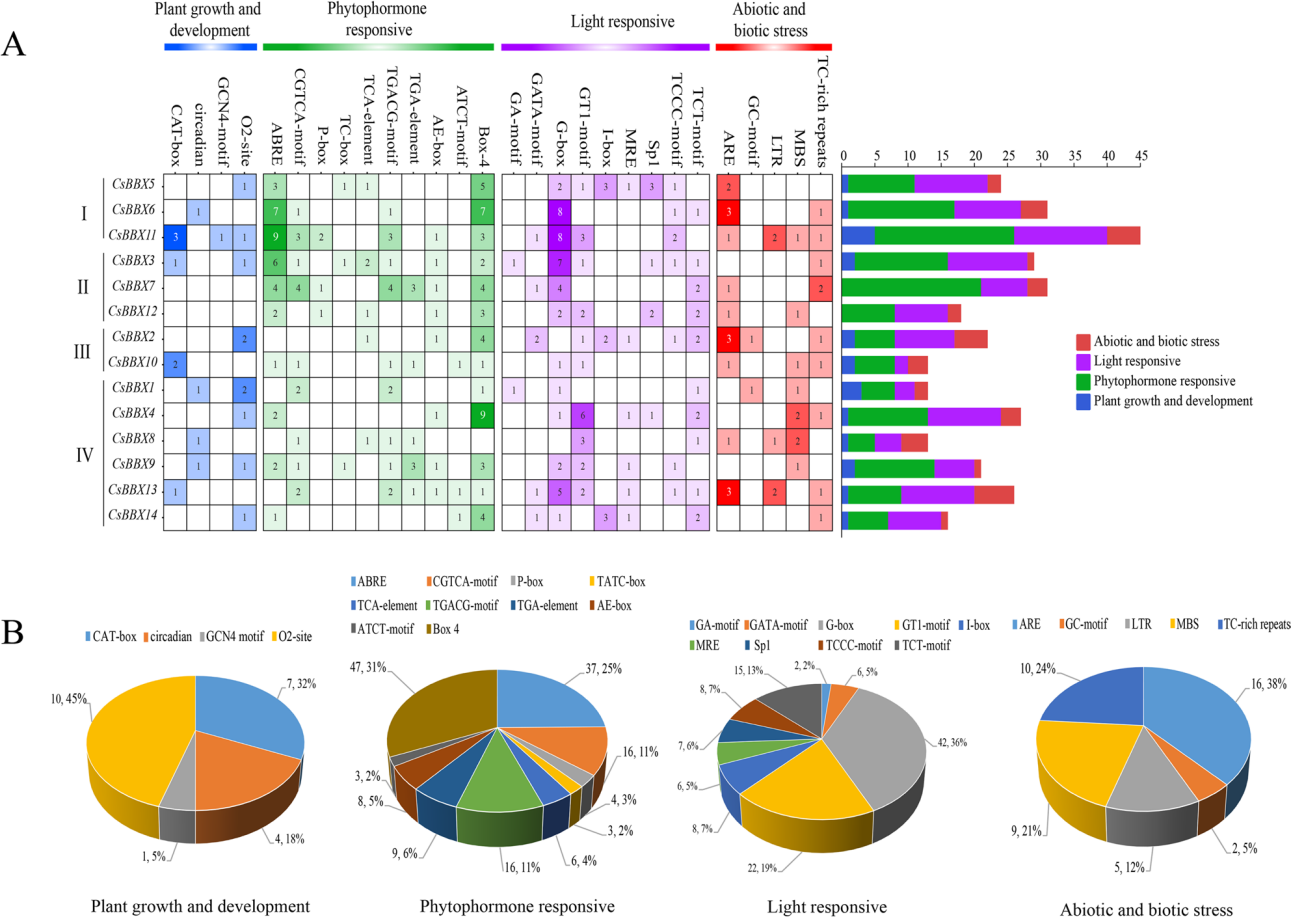
Cis-acting elements of the sweet orange *BBX* genes. **A** The varied colors and numerical values of the heatmap boxes reflect the quantities of distinct elements present in these *CsBBX* genes. Specifically, darker shades signify a greater abundance of the corresponding elements. Additionally, the histogram shows the quantity of cis-acting elements found within each *CsBBX* gene. **B** Pie chart illustrating the distribution of each promoter element across the different categories.

In conclusion, in addition to light-responsive elements, 64.75% of the cis-acting elements within promoters play crucial roles in regulating gene expression and metabolic processes in plants. The analysis of these promoter cis-acting elements suggested that the expression of the *CsBBX* genes may significantly contribute to improving the adaptive potential of sweet orange plants in the face of various adverse circumstances.

### Chromosome location and covariance analysis

**Fig. 4:**
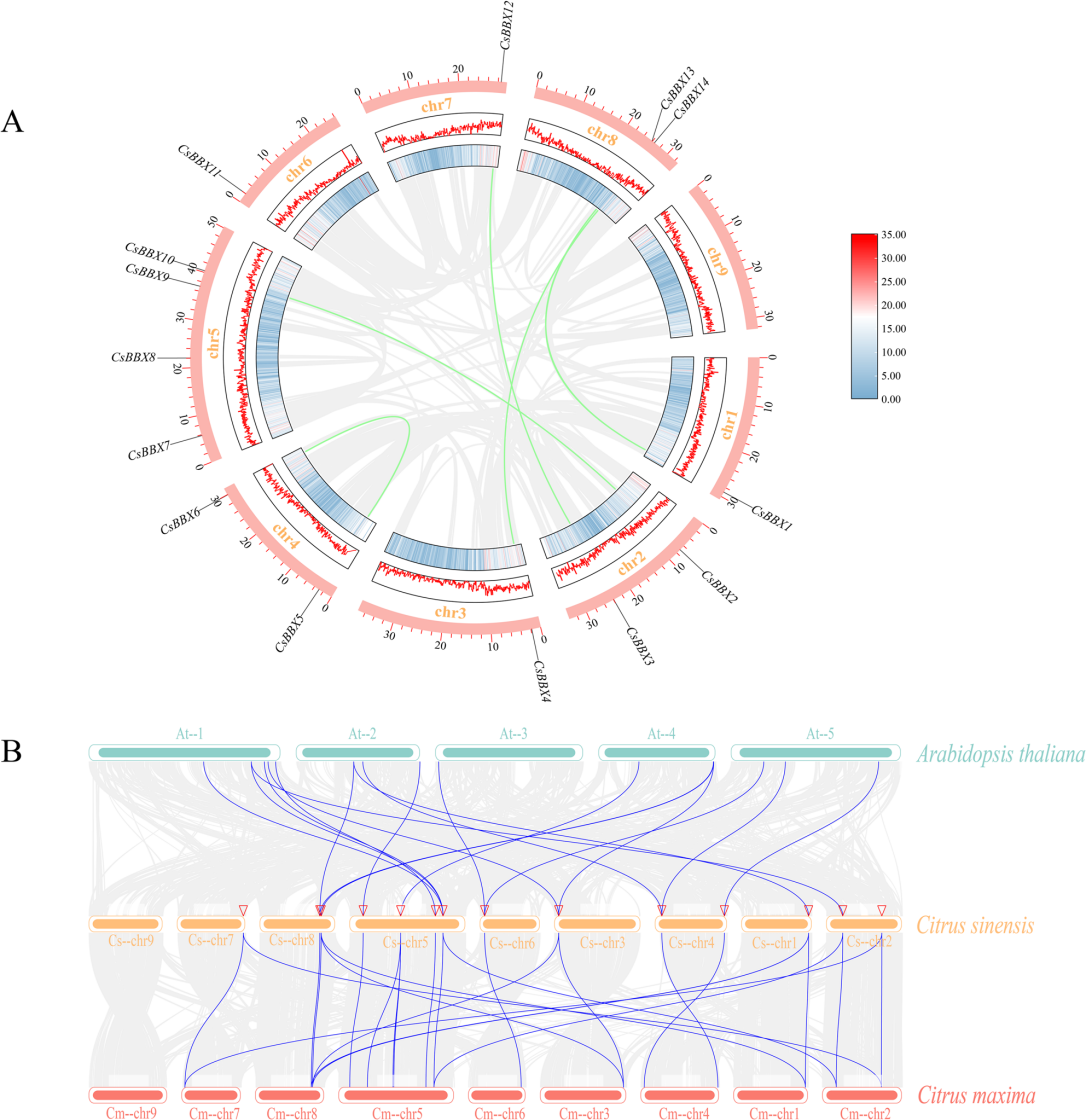
Chromosomal location and covariance analysis of sweet orange *BBX* genes. **A** From inside to outside, the first circle is the covariance distribution circle, the gray line indicates all the duplicated genes in the sweet orange genome, and the green line indicates the duplicated genes in the sweet orange *BBX* fragment. The second and third circles are heatmaps and line plots of the gene density distribution, respectively. The fourth circle shows the distribution of sweet orange *BBX* genes on the chromosomes, with the outermost markers indicating the gene names. **B** Collinearity between different species of sweet orange, *A. thaliana*, and *V. vinifera*. The gray lines indicate duplicated blocks, whereas the blue lines indicate pairs of colocalized *BBX* genes. Chromosome numbers are shown above or below each chromosome, with At, Cs, and Ci denoting chromosomes of *A. thaliana*, *Citrus sinensis*, and *Citrus maxima*, respectively

The chromosome location was identified via TBtools to analyze the chromosomal distribution of the genes and assess the genome density (Fig. 4A). The results revealed that four of the 14 genes in sweet orange were predominantly present on chromosome five (chr5), whereas no *BBX* gene was present on chromosome nine (chr9). These findings suggest that the distribution of *BBX* genes in sweet oranges is uneven across chromosomes. Furthermore, chromosome five may serve as a crucial reservoir of genetic resources for *BBX* genes in sweet oranges. In addition, there were no tandemly duplicated genes among the Cs*BBX* genes or only five segmentally duplicated gene pairs (*CsBBX1–CsBBX13*, *CsBBX2–CsBBX10*, *CsBBX3–CsBBX12*, *CsBBX4–CsBBX14*, and *CsBBX5–CsBBX6*). Segmental duplications were found for ten of the 14 *BBX* genes in sweet orange, accounting for 71.43% of the total *CsBBX*. These findings suggest that segmental duplications have played a significant role in the evolution of the *CsBBX* gene family, facilitating the emergence of new functional genes and contributing to the expansion of gene family members. Covariance analysis of sweet orange, *A. thaliana*, and *Citrus maxima* (Fig. 4B) revealed 12 and 14 orthologous pairs of *CsBBX* genes in sweet orange and the type species *A. thaliana* and in sweet orange and the closely related species *Citrus maxima*, respectively. These findings indicate that sweet orange *BBX* genes are slightly more common on the chromosomes of the closely related species *Citrus maxima* than on the chromosomes of the type species *A. thaliana*, but the difference is not very strong.

### Gene ontology (GO) function enrichment

**Fig. 5:**
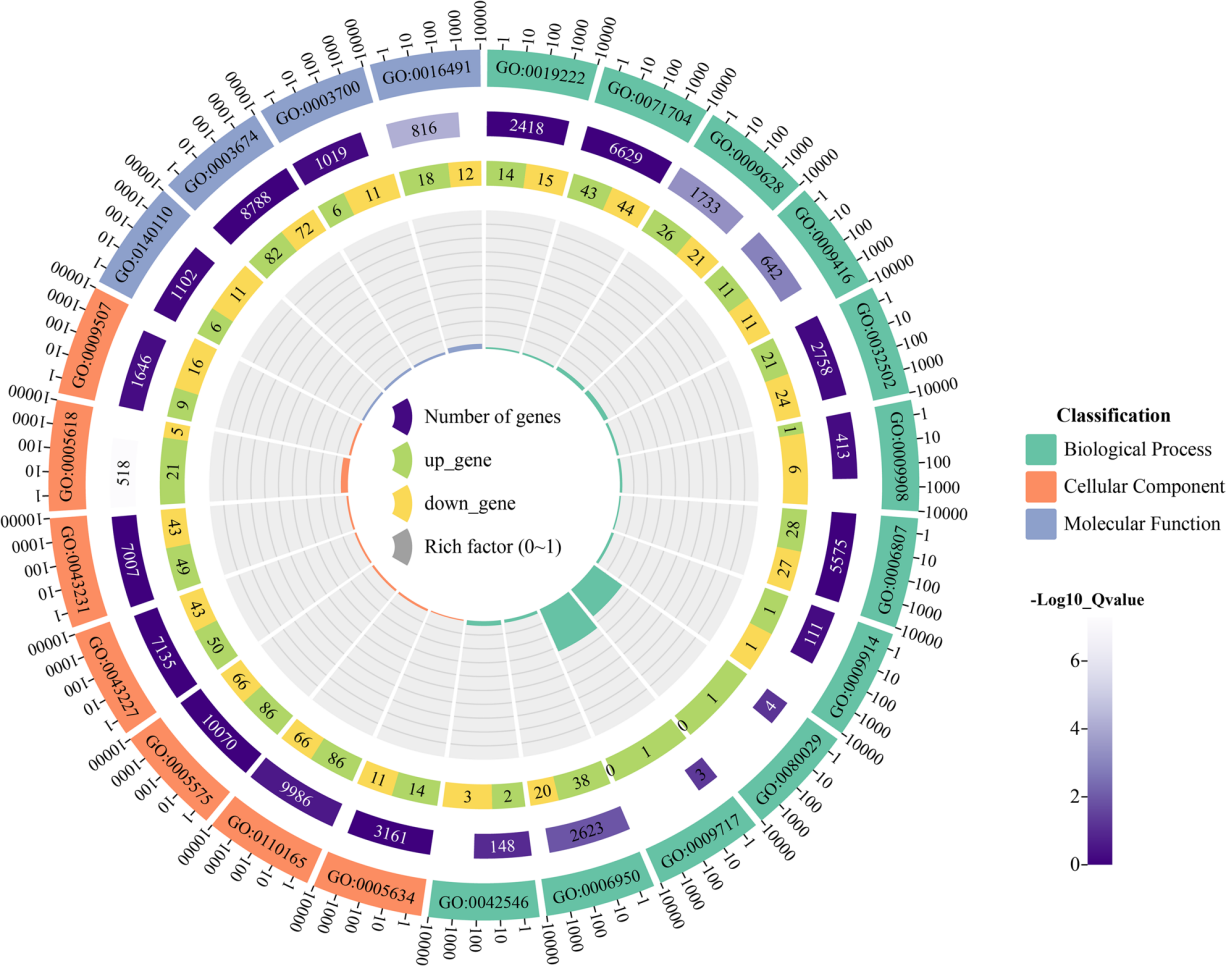
GO functional enrichment under B deficiency stress in sweet orange. The innermost circle bar represents the ratio of differentially expressed genes (DEGs) enriched in a specific Gene Ontology (GO) term compared with the total number of genes. The 2nd circle represents the number of genes enriched in the GO terms upregulated and downregulated. Green indicates upregulated genes, and yellow indicates downregulated genes. The 3rd circle heatmap represents the total number of genes enriched in the corresponding GO term. The 4th circle represents the GO number, and biological processes, cellular components, and molecular functions are indicated by different colors

When subjected to adverse conditions, plants increase their adaptability to the environment by persistently adjusting their gene expression and metabolic processes. The differentially expressed genes (DEGs) were subjected to GO enrichment analysis via transcriptome data to elucidate the regulatory functions of sweet orange genes in response to boron deficiency (Fig. 5). The results revealed enrichment of the isoflavone biosynthesis process (GO: 0009717), in which the highest proportion of genes differentially expressed from the genes of interest reached 33.33% under B deficiency stress in sweet orange. These findings indicate that the metabolism of flavonoids and sweet orange B deficiency stress are closely linked. In addition, the *CsBBX2*, *CsBBX3*, *CsBBX4*, *CsBBX6*, *CsBBX7*, *CsBBX9*, *CsBBX10*, *CsBBX11*, and *CsBBX12* genes were enriched for the regulation of metabolic processes (GO: 0019222) and organic matter metabolism (GO: 0071704) in sweet orange under boron deficiency stress. These findings indicate that sweet orange *BBX* genes can regulate metabolism and gene expression under B deficiency stress, which results in plant resilience and improved adaptation to the external environment. Additionally, *CsBBX11* was significantly enriched in response to abiotic stimuli (GO: 0009628). These findings further suggest that *CsBBX11* may play an important role in boron deficiencyin sweet oranges (Supplementary Material 3).

### Expression pattern analysis of sweet orange plants under B deficiency stress

**Fig. 6:**
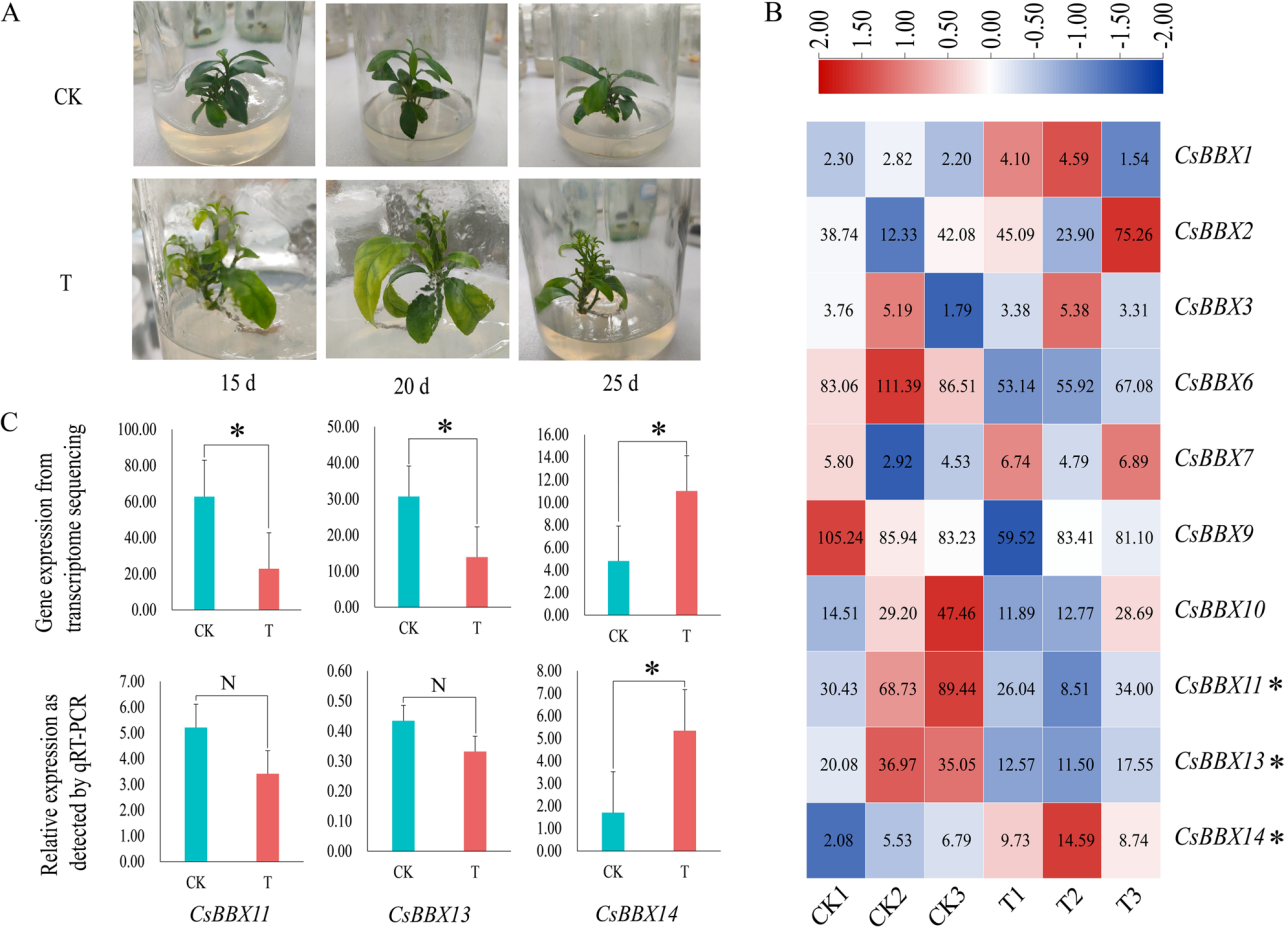
Analysis of the expression patterns of the *BBX* gene family in sweet orange under B deficiency stress. **A** Sweet orange B deficiency stress treatment. **B** Heatmap of *BBX* gene expression under B deficiency stress in sweet orange plants. **C** Transcriptome expression of the *CsBBX11*, *CsBBX13*, and *CsBBX14* genes and the RT-qPCR analysis of the corresponding genes. * indicates significant differences

To investigate the morphological characteristics of the leaves, sweet orange plants were exposed to B deficiency stress (Fig. 6A). The experimental findings indicated that the leaves of sweet orange plants subjected to boron deficiency were yellow and curled alongside swollen leaf veins. Leaf gene expression was determined via transcriptomic analysis to determine the regulatory role and expression pattern of the *BBX* gene family in response to B deficiency stress in sweet oranges (Fig. 6B). The findings indicated that 11 out of the 14 *BBX* genes in sweet orange exhibited a regulatory function under B deficiency stress; five were downregulated, and six were upregulated. *CsBBX11* and *CsBBX13* were significantly downregulated, and *CsBBX14* was significantly upregulated. These findings indicate that *CsBBX11*, *CsBBX13*, and *CsBBX14* play regulatory roles in the response of sweet orange to B deficiency stress. Furthermore, expression validation revealed that, compared with that in the CK group, *CsBBX14* expression in the T group was significantly different according to both RT-qPCR and transcriptomic analyses, with consistent trends in expression changes. These findings suggest that the *CsBBX14* gene may play a crucial role in the response of sweet orange plants to B deficiency stress (Fig. 6C).

### Analysis of transcripts and metabolites in sweet orange under B deficiency stress

**Fig. 7:**
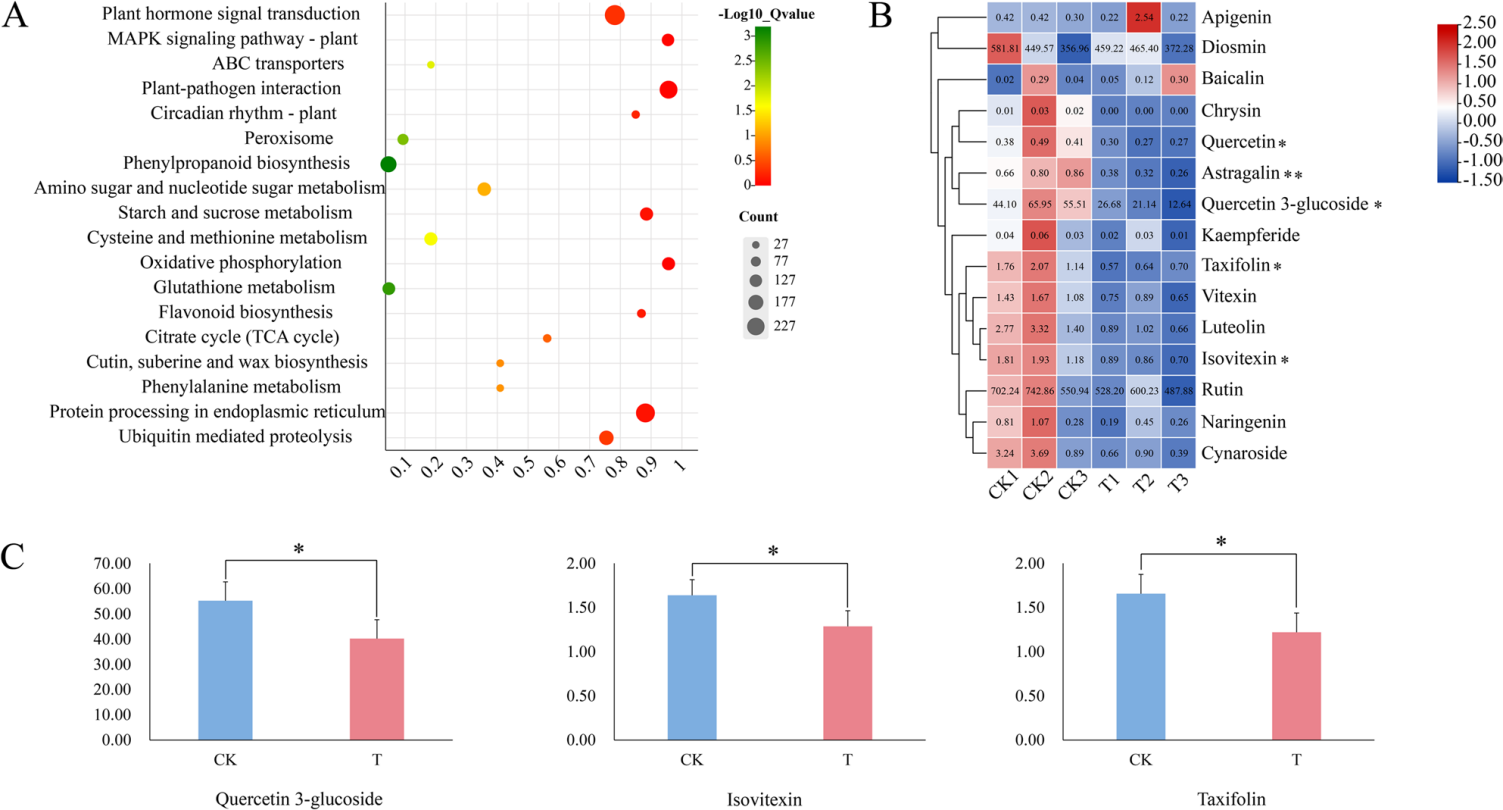
Analysis of metabolites under B deficiency stress in sweet orange. **A** KEGG metabolic pathway analysis of the DEGs under conditions of B deficiency stress in sweet orange. **B** Heatmap of the metabolite contents under B deficiency stress in sweet orange. **C** Histogram of differentially expressed metabolites under B deficiency stress in sweet orange. An * signifies a statistically significant difference at *P* < 0.05, whereas a double ** denotes a highly significant difference at *P* < 0.001

To investigate the biological functions of the DEGs in sweet orange under B deficiency stress, a KEGG enrichment analysis was performed on these genes in this study (Fig. 7A). The results revealed that 276 DEGs were enriched in plant hormone signal transduction, 198 DEGs were enriched in phenylpropanoid biosynthesis, 131 DEGs were enriched in glutathione metabolism, 54 DEGs were enriched in flavonoid biosynthesis, and 43 DEGs were enriched in the circadian rhythm-plant pathway. These findings indicate that the *BBX* gene in sweet orange may participate in the response to B deficiency stress through the aforementioned metabolic pathways. In addition, glutathione metabolism and flavonoids can help cells maintain normal immune system function, and they can promptly scavenge free radicals produced by plants in response to adverse conditions through antioxidant effects [26]. The KEGG enrichment results also indicated that in response to B deficiency stress, sweet orange genes can be regulated by regulating glutathione metabolism and flavonoid synthesis, thus preventing oxidative damage and increasing resistance to B deficiency stress.

Flavonoids can reduce oxidative stress and improve plant resistance through antioxidant signaling pathways. The contents of flavonoid metabolites were determined the basis of targeted secondary metabolomics under B deficiency stress in sweet oranges (Fig. 7B) to understand the role of flavonoids in B deficiency stress regulation in sweet oranges. The metabolites with significant differences and contents greater than one were plotted as bar graphs (Fig. 7C). Fifteen flavonoid metabolites were detected under B deficiency stress in sweet oranges. The difference in Astragalin expression was highly significant. The levels of quercetin, quercetin 3-glucoside, taxifolin, and isovitexin were significantly decreased. These findings suggest that flavonoids play a vital role in the B deficiency stress response in sweet orange plants. Furthermore, correlation analysis revealed that the potential gene *CsBBX14* is positively correlated with quercetin 3-glucoside and isovitexin but negatively correlated with taxifolin (Supplementary Material 4).

### Differentially expressed genes related to flavonoid biosynthesis in sweet orange

**Fig. 8:**
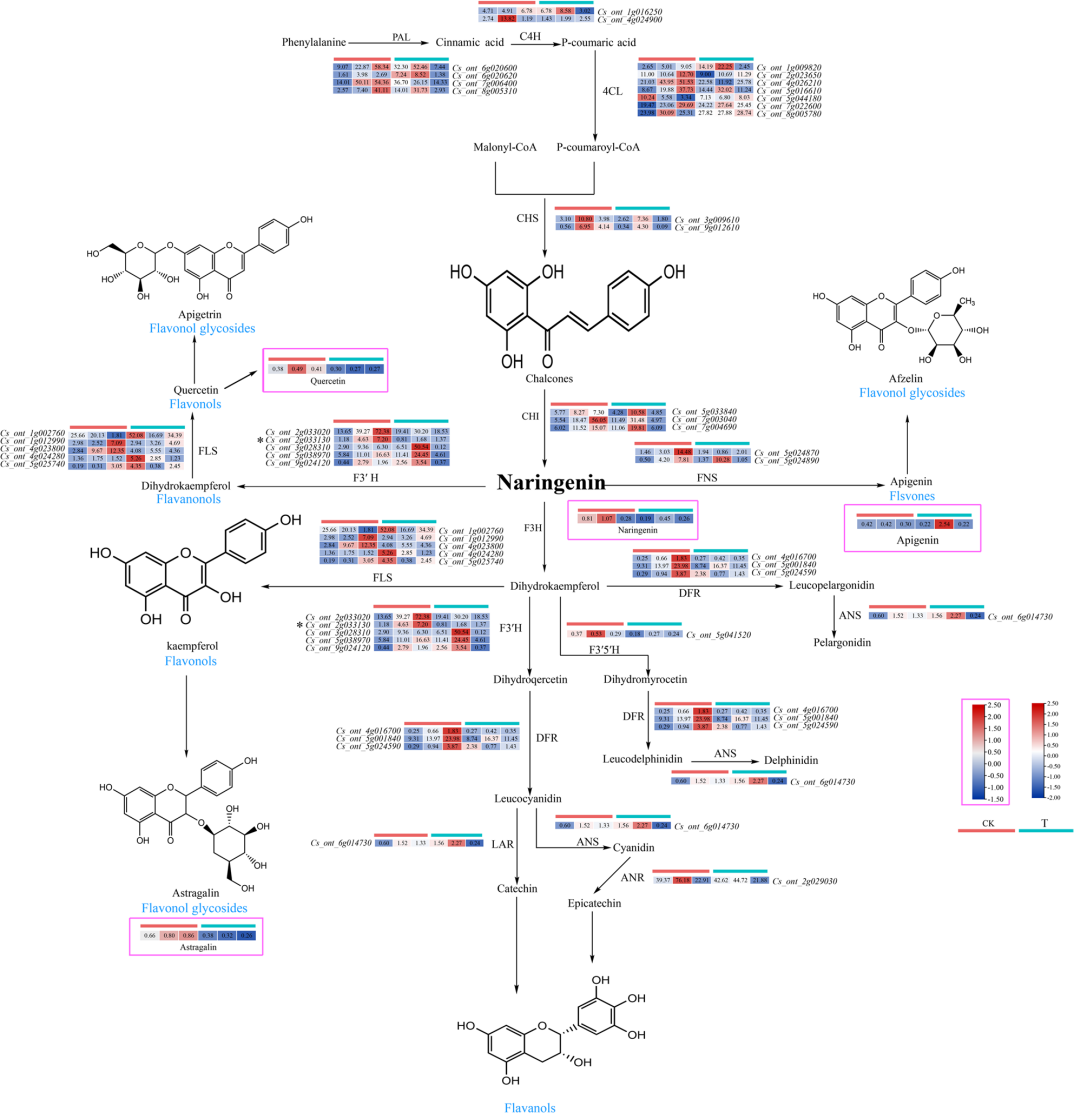
Flavonoid biosynthetic pathways in sweet orange leaves under B deficiency stress. The heatmaps in the figure indicate the expression of relevant genes and metabolites. The heatmaps with rosy red boxes indicate the metabolite content and the corresponding legends, and those without rosy red boxes indicate the expression of related genes and the corresponding legends. Blue indicates low expression (low content), and red indicates high expression (high content). PAL, phenylalanine ammonia-lyase. C4H, cinnamate 4-hydroxylase. 4CL, 4-coumaroyl CoA ligase. CHS, chalcone synthase. CHI, chalcone isomerase. FNS, flavanone synthase. F3H, flavanone 3b-hydroxylase. F3′H, Flavonoid 3′-hydroxylase. F3′5′H, Flavonoid 3′,5′-hydroxylase. FLS, flavonol synthase. LAR, leucoanthocyanidin reductase. ANS, Anthocyanin synthase. ANR, Anthocyanin reductase. The red and turquoise lines indicate the control (CK) and boron deficiency (T) treatment groups, respectively. The heatmap in the rosy red box indicates the distribution of flavonoid metabolite content, and the rest of the heatmap shows the distribution of expression of sweet orange-related genes

Changes in flavonoid biosynthesis in sweet orange leaves under B deficiency stress were investigated via combined transcriptomic and metabolomic analyses (Fig. 8). A total of fifty-five genes related to sweet oranges, along with four flavonoids, were identified as playing significant roles in the flavonoid biosynthesis pathway. P-coumaroyl-CoA synthesis pathway: Thirteen genes (four *PAL*, two *C4H*, and seven *4CL* genes) were involved. Among these genes, six genes were upregulated, whereas seven genes were downregulated. In the naringenin synthesis pathway, five genes (two *CHSs* and three *CHIs*) were involved; four genes were downregulated, and one gene was upregulated. Overall, these genes tended to be downregulated, which is consistent with the trend in the change in the naringenin content. In the quercetin synthesis pathway, 15 genes (two *CHSs*, three *CHIs*, five *F3′Hs*, and five *FLSs*) were involved. Among them, eight genes were downregulated, and seven genes were upregulated. *F3′H* (*Cs_ont_2g033130*) was significantly downregulated according to gene variance, which is consistent with the trend observed for quercetin content. Seven genes (two *CHS*, three *CHI*, and two *FNS* genes) were involved in the apigenin synthesis pathway. Five genes were downregulated, and two were upregulated, showing an overall trend toward downregulation, which is consistent with the change in apigenin (apigenin) content. In the astragalin synthesis pathway, ten genes (two *CHSs*, three *CHIs*, and five *FLSs*) were involved. Six genes were downregulated, and four were upregulated, indicating an overall downregulation trend. These findings were in line with the observed variations in Astragalin content. Within the entire flavonoid biosynthetic pathway, 54% of the genes presented a decreasing trend. Notably, the expression of the *Cs_ont_2g033130* gene was significantly downregulated during the biosynthesis of dihydrokaempferol. These findings indicate that sweet orange genes play a prime regulatory role in the B deficiency stress response. The flavonoid contents of sweet orange plants under B deficiency stress tended to decrease. The above changes in gene expression and metabolite content indicate that sweet oranges can respond to B deficiency stress by regulating the synthesis of flavonoids through their gene expression regulation to improve their adaptability to the environment.

## Discussion

The primary global production regions for sweet oranges are concentrated in China, Brazil, and the United States. Within China, the major planting areas are found in the provinces of Sichuan, Hubei, Hunan, Guangdong, and Yunnan. As the cultivation area of citrus continues to expand, B deficiency has become increasingly prevalent. 50% of the effective B content of citrus plantation soil was deficient in Yuxi, Yunnan. It severely affects sweet orange fruit yield and economic value. Therefore, selecting and breeding sweet orange varieties resistant to B deficiency stress is practical. *BBX* genes are vital for the regulation of boron homeostasis in plants [6]. Flavonoids are compounds that effectively scavenge excessive reactive oxygen species generated by stress within plants. *BBX* and flavonoid-related genes can effectively improve plant resistance. Nonetheless, the precise mechanisms through which both factors influence the response of sweet orange plants to boron deficiency remain unclear. Therefore, through transcriptomic and RT-qPCR analyses, this study revealed that *CsBBX14* may play an essential role in the response of sweet orange to B deficiency stress. Correlation analysis revealed that *CsBBX14* is closely associated with key metabolites (quercetin 3-glucoside, taxifolin, and isovitexin) as well as sweet orange genes that affect these metabolites. These findings provide valuable insights for the selection and breeding of sweet orange varieties resistant to B deficiency.

A phylogenetic tree was constructed from the sweet orange *BBX* gene family, which included 14 family members, via multiple sequence comparisons of its gene sequences with those of the *A. thaliana*, *V. vinifera*, rice, *S. lycopersicum*, *P. bretschneideri*, and *M. pumila BBX* genes. Several *BBX* genes were classified according to the classification of *A. thaliana BBX* genes. The results revealed that the *BBX* genes of sweet orange were organized into four distinct subclades and that all the other species were classified into five subclades. These findings suggest that sweet orange has evolved similarly to several other species without significant differences. Rajnish and Zhang reported that there are 31 members and five subclades in the *BBX* gene family of *A. thaliana* [27]. Moreover, the *BBX* gene family of *V. vinifera* has 25 members and is classified into five subclades. The gene family of *V. vinifera* comprises 25 distinct members, which are categorized into five subclades [28]. This observation aligns with the results presented in this study. In the constructed phylogenetic tree, 57% of the genes associated with sweet orange presented clustering patterns on the same branch as those associated with *A. thaliana*, and no genes were clustered on the same branch as those associated with rice. Furthermore, the phylogeny of sweet oranges was more similar to that of dicotyledonous plants, especially *A. thaliana*, than to that of monocotyledonous plants. In addition, while the number of subfamilies for each species sightly varied, the quantity of genes within each subfamily differed significantly among the species. This disparity can be attributed primarily to variations in the identified gene families, as well as differences in their origins and evolutionary trajectories among species. *V. vinifera BBX* gene family members were identified via HMM model comparison and SMART conserved structural domain validation, and the E value reached 0.01. However, the *BBX* gene family members of sweet orange in the present study were identified by combining the HMM and BLAST comparisons, and the E value was less than 10^− 5^. The prediction of *BBX* gene family members in sweet oranges was conducted with a much higher level of stringency than that in *V. vinifera*, resulting in higher reliability. Therefore, the number of sweet orange *BBX* genes was lower than the *V. vinifera BBX* gene number. *A. thaliana AtBBX21*, *AtBBX22*, and *AtBBX23* directly and positively affect plant photomorphogenesis and flavonoid accumulation [29]. In contrast, *CsBBX14*,* AtBBX21*, *CsBBX9*,* AtBBX22*, *CsBBX8*, and *AtBBX23* were located on the same branch of the phylogenetic tree and presented self-expansion values exceeding 90%. These findings suggested that sweet orange *CsBBX8*, *CsBBX9*, and *CsBBX14* may promote photomorphogenesis and flavonoid synthesis. The overexpression of *AtBBX22* led to increased anthocyanin accumulation in *A. thaliana* plants, whereas *AtBBX21* and *AtBBX23* play regulatory roles in the synthesis of anthocyanins under light conditions [30]. In *M. pumila*, the interaction between *MdBBX22* and *MdHY5* enhances the expression of *MdMYB1*, a key regulator that promotes the accumulation of anthocyanins [31]. In the phylogenetic tree, the *CsBBX9* gene was clustered in the same branch as the *AtBBX22* and *MdBBX22* genes, and the *CsBBX14* gene was clustered in the same branch as the *AtBBX21* gene, exhibiting bootstrap values exceeding 90%. These results indicate that *CsBBX9* and *CsBBX14* may be involved in the regulatory mechanisms governing anthocyanin synthesis.

Gene duplication serves as a mechanism for gene amplification and represents one of the primary molecular processes through which genes undergo evolution to generate novel functions [29]. Chromosomal location and covariance analyses were performed on the *BBX* gene family of sweet oranges. The findings demonstrated that the 14 *BBX* genes in sweet orange were located across eight chromosomes, with an absence of *BBX* gene distribution observed on chromosome nine. These findings indicated that the distribution of *BBX* genes in sweet oranges was uneven across chromosomes. In addition, five pairs of fragment duplications were detected in the sweet orange *BBX* genes. The number of fragment duplication genes accounted for 71.43% of the number of sweet orange *BBX* genes. These findings illustrate that the sweet orange *BBX* gene may contain vital genetic resources for new functional gene generation and gene family member expansion. A study of *MYB* genes in gooseberry revealed that 24% of *MYB* genes are derived from segmental duplication [32]. These findings further suggest that gene duplication events are vital pathways for new gene generation.

In response to environmental stress, plant cells initiate gene expression programs that govern the accumulation of metabolites, facilitating their adaptation to altered environmental conditions. Transcriptome analysis revealed the molecular mechanism underlying the changes in flavonoid content in sweet orange leaves under B deficiency stress in this study. Moreover, GO annotation and KEGG enrichment analyses revealed that sweet orange genes play vital regulatory roles in the accumulation and transport of flavonoids under B deficiency stress. Excessive ROS and oxidative free radicals are produced when plants are subjected to external environmental stresses. At this time, plants regulate the accumulation and transport of flavonoids in vivo through gene expression, thereby increasing their ability to scavenge oxidative free radicals and reduce cellular oxidative damage. The activity of scavenging reactive oxygen species is closely associated with both the quantity and the placement of hydroxyl groups present on the benzene ring. For example, quercetin predominantly engages with free radicals via its phenolic OH groups, leading to the formation of semiquinone radicals, which can effectively interrupt the chain reactions of free radicals. A new stable group is formed, and the oxidation reaction is interrupted or delayed when a substance loses electrons or a hydrogen supply during its reaction with free radicals [33]. In the present study, we investigated the expression of key rate-limiting genes associated with naringenin, quercetin, apigenin, and astragalin synthesis, which tended to increase overall. However, the flavonoid content decreased under conditions of B deficiency stress in sweet oranges. These findings indicate that sweet orange consumes flavonoids in its own body, effectively removing excessive reactive oxygen species and oxidative free radicals generated by stress in the body. This result aligns with the findings from earlier analyses. However, a study on magnesium stress in sweet oranges reported that the content of flavonoid metabolites increased under magnesium stress, which is inconsistent with the findings of the present study [34]. This discrepancy may stem from variations in the stress treatment stage of the plants at the time of sampling; however, the specific cause requires further verification.

**Fig. 9:**
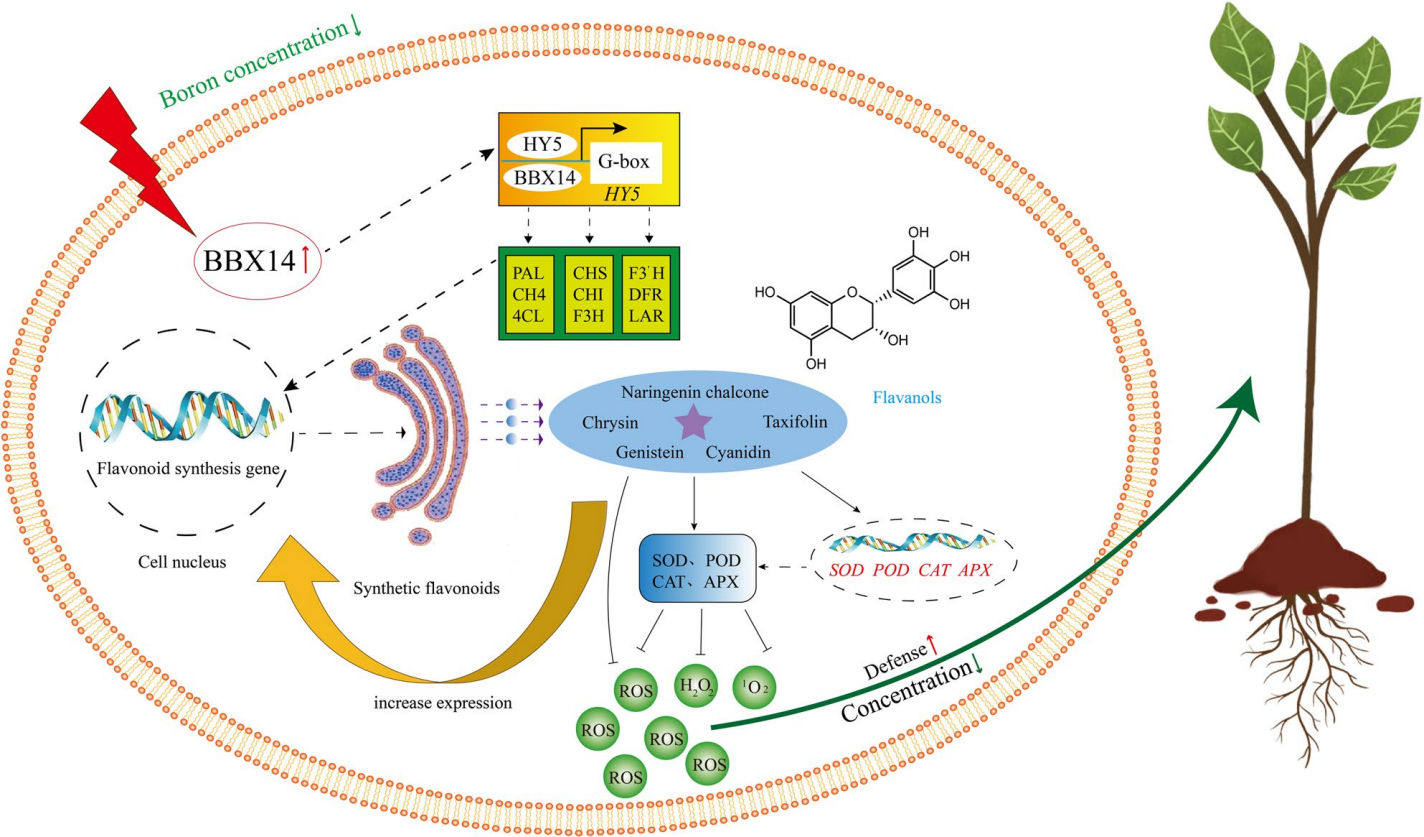
Model of the regulatory effect of B deficiency stress on sweet orange

Environmental factors play a significant role in regulating gene expression by influencing transcription factors that specifically associate with the promoters of target genes. Within this context, the *BBX* gene family has been demonstrated to be crucial in modulating gene expression, particularly in the flavonoid biosynthetic pathway [35]. *MdBBX22* induces the activation of the promoters of *MdANR* and *MdLAR* in *M. pumila* via mdm-miR 858, which promotes the biosynthesis of the flavonoid proanthocyanidin [36]. The proteins *PpBBX18* and *PpBBX21* are known to interact with *PpHY5*, playing crucial roles in the regulation of anthocyanin biosynthesis in *Prunus persica* [37]. The *AtBBX21* gene can directly interact with HY5 to increase *HY5* gene expression, and the *AtBBX21* gene can bind to the G-box element found within the HY5 promoter, thereby activating its transcription and facilitating the expression of genes involved in flavonoid synthesis [38]. This enhances the ability of flavonoids such as taxifolin to scavenge ROS to some extent, which in turn reduces oxidative damage in plants. In this study, phylogenetic analysis revealed that the *CsBBX14* and *AtBBX21* genes clustered within the same clade, with bootstrap values greater than 90%. These findings suggest that the *CsBBX14* gene may exhibit functional similarity to the *AtBBX21* gene. Furthermore, correlation analysis revealed a highly significant correlation (57%) between *CsBBX14* and the genes involved in flavonoid biosynthesis and metabolism in sweet orange (Supplementary Material 5). Based on our findings, we propose a mechanistic model in which the *CsBBX14* gene positively regulates flavonoid functionality, ultimately conferring enhanced stress resistance to plants (Fig. 9). When the concentration of B in the environment decreases, *CsBBX14* may interact with the G-box element located in the promoter of HY5, thus initiating its transcription and subsequently increasing the expression of genes involved in flavonoid synthesis [39]. Flavonoids can effectively inhibit the production of reactive oxygen species in stressful environments, reduce oxidative damage in plant cells, and improve stress tolerance. *CsBBX14* expression was significantly elevated in this study. These results indicate that this gene could be highly involved in the plant response to boron deficiency by influencing the synthesis of flavonoids. However, the specific function of the *CsBBX14* gene still needs further verification.

In conclusion, these findings provide a vital reference for enhancing the resistance of sweet orange plants and improving the quality of sweet orange fruits from a molecular perspective. However, there are several limitations in this study, and the potential role of *CsBBX14* in the synthesis of flavonoids still needs to be verified by more specific experiments, such as gene knockdown. In the future, we will perform further in-depth studies of this gene.

## Conclusions

Transcriptomic analysis and RT-qPCR expression validation revealed that *CsBBX11*, *CsBBX13*, and *CsBBX14* play important regulatory roles in the response of sweet orange to boron (B) deficiency stress. In particular, *CsBBX14* is likely the gene that performs crucial functions. Metabolomic analysis revealed that quercetin 3-glucoside, taxifolin, and isovitexin are important metabolites involved in the response of sweet orange to boron deficiency stress. Notably, *CsBBX14* was found to be closely associated with these potential metabolites. In addition, we propose a model of the regulatory effect of B deficiency stress on sweet orange. These findings offer fresh insights into enhancing the stress tolerance of sweet orange plants.

## Supplementary Information

Supplementary Material 1.

Supplementary Material 2.

Supplementary Material 3.

Supplementary Material 4.

Supplementary Material 5.

## Data Availability

All the data generated or analyzed during this study are included in this published article. The C. sinensis genome (v3.0) and annotation files of sweet orange are openly available in the CPBD: Citrus Pangenome to Breeding Database (http://citrus.hzau.edu.cn/index.php). RNA-Seq data under biotic and abiotic stress conditions can be found under accession numbers GSE249048 (https://www.ncbi.nlm.nih.gov/geo/query/acc.cgi? acc=GSE249048). The sample IDs are GSM7925570, GSM7925571, GSM7925572, GSM7925573, GSM7925574, and GSM7925575. The RNA-Seq data are publicly available at the National Center for Biotechnology Information. The other data presented in this study are available in the Supplementary Materials.
