# Supplementary figures and images for: Transcription-metabolism association analysis of molecular mechanisms in sweet orange plants in response to boron deficiency
Source: BMC Genomics. 2025 Dec 22;26:1136. doi: 10.1186/s12864-025-12408-w (PMC12752163; doi:10.1186/s12864-025-12408-w)

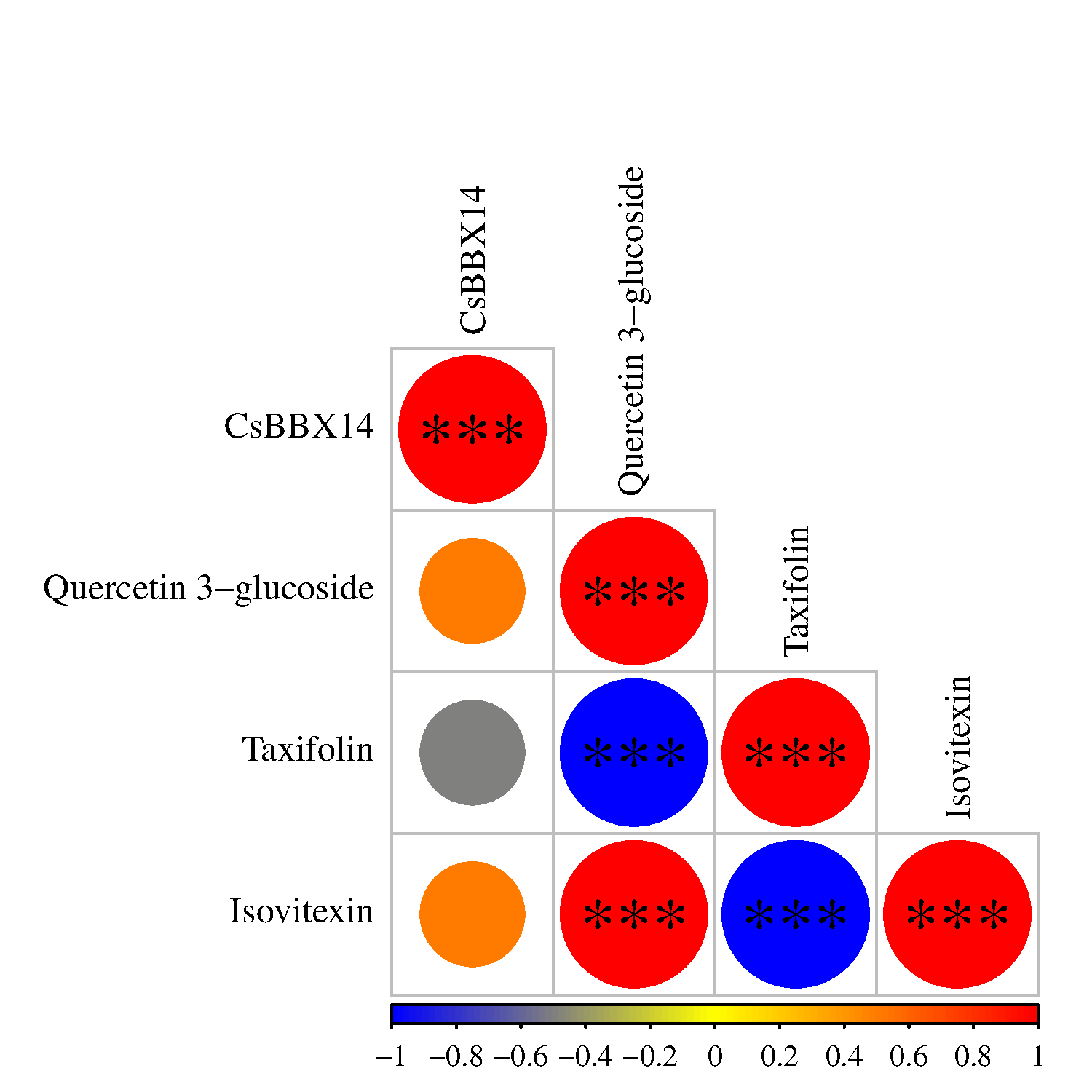

Supplement: Supplementary file 4 — Supplementary Material 4. [file 12864_2025_12408_MOESM4_ESM.png]

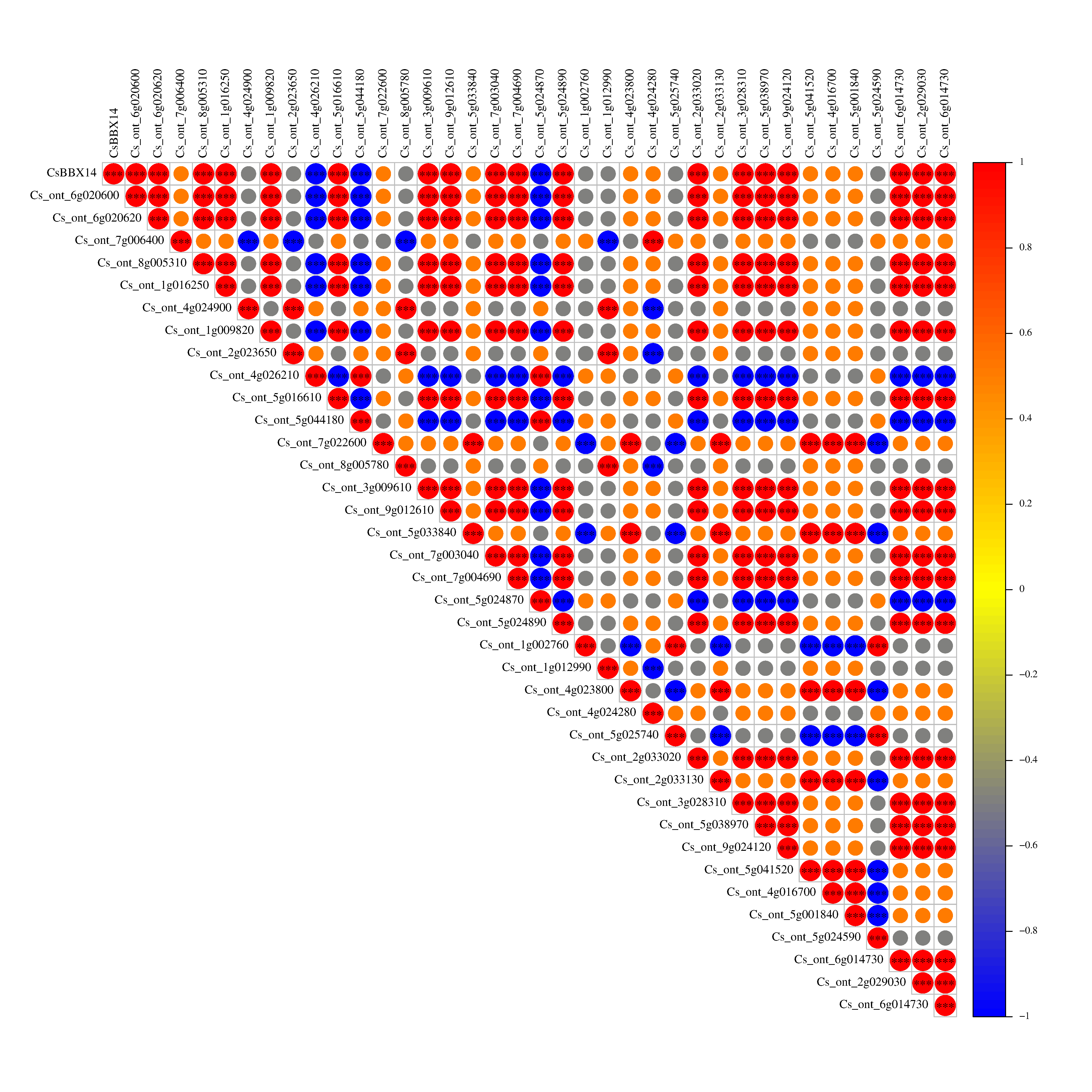

Supplement: Supplementary file 5 — Supplementary Material 5. [file 12864_2025_12408_MOESM5_ESM.png]
